# PP45 Budget Impact Analysis Of Including Bictegravir/Emtricitabine/Tenofovir Alafenamide In First-Line Antiretroviral Therapy For HIV/AIDS In Chile’s Explicit Health Guarantees

**DOI:** 10.1017/S026646232510264X

**Published:** 2025-12-29

**Authors:** Daniela Sugg, Maximiliano Alarcón, Dino Sepúlveda, Moisés Russo

## Abstract

**Introduction:**

HIV/AIDS continues to pose a significant public health challenge in Chile, despite advances in prevention and treatment. The Explicit Health Guarantees (GES) framework provides comprehensive access to antiretroviral therapy (ART) for eligible patients. This study assessed the budget impact of including bictegravir/emtricitabine/tenofovir alafenamide (BET) in first-line ART regimens for treatment-naïve adults under the Ministry of Health methodology for the GES program, evaluating its clinical benefits and financial viability.

**Methods:**

A budget impact model was developed from the perspective of the GES system, utilizing updated public data on ART costs, therapy utilization, and demographic projections for 2025 to 2027. The analysis incorporated updated ART prices, therapy utilization frequencies, and clinical efficacy evidence. BET was evaluated as an alternative to abacavir/dolutegravir/lamivudine (ADL) using comparative evidence from clinical studies. Three scenarios were modeled: current prices, adjusted usage frequencies, and inclusion of BET in the treatment framework.

**Results:**

The inclusion of BET had no significant budget impact, primarily due to cost-saving efficiencies from centralized procurement by the National Health Supply Center. Updated ART prices for 2023 indicated a 34.7 percent reduction compared with the 2022 Verification of Costs Study. Projected costs for GES HIV/AIDS management, including BET, were USD259 million (2025), USD284 million (2026), and USD312 million (2027). Therapy uptake and procurement efficiencies were the primary cost drivers. The addition of BET enhanced therapeutic options without imposing additional financial burden.

**Conclusions:**

The integration of BET into first-line ART regimens aligns with the GES goals of financial sustainability and clinical efficacy, offering an effective alternative for HIV management. These findings underscore the importance of evidence-based policymaking and optimized resource allocation to improve healthcare outcomes in Chile.

